# Chronic symptoms in patients with unilateral vestibular hypofunction: systematic review and meta-analysis

**DOI:** 10.3389/fneur.2023.1177314

**Published:** 2023-07-07

**Authors:** Mustafa Karabulut, Lien Van Laer, Ann Hallemans, Luc Vereeck, Vincent Van Rompaey, Wolfgang Viechtbauer, Ali Melliti, Lisa van Stiphout, Alfarghal Mohamad, Angélica Pérez Fornos, Nils Guinand, Raymond van de Berg

**Affiliations:** ^1^Division of Balance Disorders, Department of Otorhinolaryngology and Head and Neck Surgery, School for Mental Health and Neuroscience, Maastricht University Medical Center, Maastricht, Netherlands; ^2^Department of Rehabilitation Sciences and Physiotherapy/Movant, Faculty of Medicine and Health Science, University of Antwerp, Antwerp, Belgium; ^3^Department of Otorhinolaryngology and Head & Neck Surgery, Antwerp University Hospital, Faculty of Medicine and Health Sciences, University of Antwerp, Antwerp, Belgium; ^4^Department of Psychiatry and Neuropsychology, Maastricht University, Maastricht, Netherlands; ^5^Department of Ear Nose Throat, King Abdul Aziz Medical City, Jeddah, Saudi Arabia; ^6^Service of Otorhinolaryngology Head and Neck Surgery, Department of Clinical Neurosciences, Geneva University Hospitals, Geneva, Switzerland

**Keywords:** unilateral vestibular hypofunction, unilateral vestibulopathy, unilateral vestibular loss, unilateral vestibular areflexia, chronic symptoms, dizziness, imbalance, vertigo

## Abstract

**Objective:**

To systematically evaluate the full spectrum of self-reported chronic symptoms in patients with unilateral vestibular hypofunction (UVH) and to investigate the effect of interventions on these symptoms.

**Methods:**

A systematic review was conducted following the guidelines of the Preferred Reporting Items for Systematic Review and Meta-Analysis Statement (PRISMA). A literature search was performed in Pubmed, Web of Science, Embase, and Scopus to investigate self-reported symptoms and self-report questionnaires in patients with UVH. All original studies ranging from full-text clinical trials to case reports, written in English, German, and French, were included. The frequency of self-reported symptoms was presented. For self-report questionnaires, a meta-analysis was carried out to synthesize scale means by the pre- and post-intervention means and mean changes for studies that investigated interventions.

**Results:**

A total of 2,110 studies were retrieved. Forty-seven studies were included after title-abstract selection and full-text selection by two independent reviewers. The symptoms of UVH patients included chronic dizziness (98%), imbalance (81%), symptoms worsened by head movements (75%), visually induced dizziness (61%), symptoms worsened in darkness (51%), and oscillopsia (22%). Additionally, UVH could be accompanied by recurrent vertigo (77%), tiredness (68%), cognitive symptoms (58%), and autonomic symptoms (46%). Regarding self-report questionnaires, UVH resulted on average in a moderate handicap, with an estimated mean total score on the Dizziness Handicap Inventory (DHI) and the Vertigo Symptom Scale (VSS) of 46.31 (95% CI: 41.17–51.44) and 15.50 (95% CI: 12.59–18.41), respectively. In studies that investigated the effect of vestibular intervention, a significant decrease in the estimated mean total DHI scores from 51.79 (95% CI: 46.61–56.97) (pre-intervention) to 27.39 (95% CI: 23.16–31.62) (post intervention) was found (*p* < 0.0001). In three studies, the estimated mean total Visual Analog Scale (VAS) scores were 7.05 (95% CI, 5.64–8.46) (pre-intervention) and 2.56 (95% CI, 1.15–3.97) (post-intervention). Finally, a subgroup of patients (≥32%) persists with at least a moderate handicap, despite vestibular rehabilitation.

**Conclusion:**

A spectrum of symptoms is associated with UVH, of which chronic dizziness and imbalance are most frequently reported. However, semi-structured interviews should be conducted to define the whole spectrum of UVH symptoms more precisely, in order to establish a validated patient-reported outcome measure (PROM) for UVH patients. Furthermore, vestibular interventions can significantly decrease self-reported handicap, although this is insufficient for a subgroup of patients. It could therefore be considered for this subgroup of patients to explore new intervention strategies like vibrotactile feedback or the vestibular implant.

**Systematic review registration:**

[https://www.crd.york.ac.uk/prospero/], identifier [CRD42023389185].

## Introduction

Unilateral vestibular hypofunction (UVH) is a heterogeneous disorder in which a partial or complete loss of one of the vestibular organs and/or nerves is present (1, 2). UVH occurs either suddenly or gradually, depending on the etiology. The reported vestibular symptoms of UVH include, among others, dizziness, imbalance, and oscillopsia (an illusion of an unstable vision) and the time course and impact of these symptoms can vary between patients (3–5). They can occur both in static conditions (no body/head movements) and in dynamic conditions (with body/head movements). In case UVH symptoms occur, a neurological process called vestibular compensation can decrease these symptoms (6, 7). However, vestibular compensation is most effective for symptoms in static conditions (8), and less effective for dynamic conditions: approximately 29–66% of UVH patients remain to have vestibular symptoms in dynamic conditions, despite vestibular rehabilitation (9–12). This can cause chronicity and results in a bunch of chronic symptoms in patients with UVH. It was previously demonstrated that a whole spectrum of additional symptoms can be related to UVH, varying from visually induced dizziness (13, 14), impaired spatial navigation and motion perception (14–17), to cognitive complaints (14, 18), autonomic complaints (14, 19), and increased tiredness (14). UVH can therefore significantly affect quality of life (20).

In the literature, different methods are used to capture chronic UVH symptoms (i.e., ≥3 months (21)): history taking (22); questionnaires like the Dizziness Handicap Inventory (DHI) (23) and Vertigo Symptom Scale (VSS) (24); scales like the Visual Analog Scale (VAS) (25); and semi-structured interviews (26). Regarding questionnaires, the DHI and VSS are commonly used patient-reported outcome measures (PROMs) in this patient population. The DHI is a validated, self-reported questionnaire composed of 25 questions to quantify the impact of dizziness on daily life. For each question, “yes,” “sometimes,” or “no” (that correspond to four, two, or zero points) are answered by patients. The DHI scores categorize the self-perception of dizziness as mild (0–30); moderate (31–60); and severe (61–100) (23, 27). The VSS, consisting of 15 items, reflects severity and frequency of dizziness symptoms within the last month. Each item is scored on a 5-point scale (range 0–4) and hence the total scale score ranges from 0 to 60. Severe dizziness is defined as ≥12 points on the total scale (28). The VAS, which can be used to assess different aspects (e.g., dizzines intensity, dizziness frequency, visual vertigo), subjectively evaluates the perception of vestibular symptoms related to vertigo, dizziness, imbalance, and oscillopsia. In the VAS, dizziness severity is categorized as none (1); slight (2–3); mild (4–5); moderate (6–7); severe (8–9); and extreme (10) (29). A semi-structured interview, which is a combination of a structured and unstructured interview, can also be performed to evaluate symptoms related to UVH. It comprises open-ended questions to facilitate subjects freely expressing their own experiences, opinions, and attitudes (30).

Each method has its pros and cons. For example, questionnaires reflect patients’ subjective experiences. This might be an advantage since it avoids interpretation by the clinician and they facilitate quantification of symptoms. On the other hand, it could be a disadvantage since a clinician might ask relevant questions which go beyond the focus of the questionnaire (31). This indicates that no method is perfect to reliably capture the whole spectrum of chronic UVH symptoms, and a combination of these methods might be preferred. In addition, various intervention methods such as vestibular rehabilitation therapy, medical management, surgery, and psychotherapy are applied to see if there is any effect on chronic symptoms in patients with UVH. Depending on the etiology, and prognosis of the disease, the method to be used varies. The effect of interventions on symptoms is mostly provided through patient-reported outcome measures.

Although many symptoms related to UVH have been described in the literature, a structured overview of chronic symptoms related to UVH is currently lacking. Therefore, the objective of this study was to systematically review the full spectrum of chronic UVH symptoms. In addition, the effect (or lack of effect) of interventions on these symptoms was evaluated. These findings could serve as the first step to establish a validated PROM specifically for patients with UVH.

## Materials and methods

### Registration and protocol

This systematic review was carried out following the guidelines of the Preferred Reporting Items for Systematic Review and Meta-Analysis Statement (PRISMA) (32). The protocol was registered by the International Prospective Register of Systematic Reviews (PROSPERO) (www.crd.york.ac.uk/prospero; registration no. CRD42021260512).

### Data sources and searches

The last systematic search was conducted on November 4, 2022, in the following databases: Pubmed, Web of Science, Embase, and Scopus. “Unilateral Vestibular Hypofunction” (Population), “Chronic Symptoms, ≥3 months (Outcome).” Specific search queries were used (see Supplementary Tables S1, S2). The search queries were developed by three of the authors (MK, LVL, RvdB), in cooperation with an independent librarian at Maastricht University. No filters were applied.

### Study selection

Using predefined inclusion and exclusion criteria (see below), possibly relevant articles were selected. All publications were first exported to EndNote X9 software. After that, the option “Find Duplicates” was applied in order to remove the publications appearing in more than one database. Two independent reviewers (MK, LVL) screened the articles first on title and abstract (stage 1) and subsequently, a complete reading of the full-text articles was performed (stage 2). Following each phase, inconsistencies regarding inclusion and exclusion criteria between the reviewers were discussed in consensus meetings. Consensus was reached for all cases. Eventually, to ensure that no relevant articles were missed, the references of the articles included after phase 2, were screened and included if eligible.

### In-and exclusion criteria

The in-and exclusion criteria can be found in the Supplementary Table S3. Regarding study design, all original studies ranging from full text clinical trials to case reports, written in English, German, and French were included. Conference abstracts/−reports, letters, abstracts only, animal studies, editorials, (systematic) reviews, and meta-analysis were excluded. Regarding the study population, only studies were selected which included adult UVH patients (≥18 years) with etiologies that could possibly lead to chronic UVH (e.g., acute unilateral vestibulopathy/vestibular neuritis, Menière’s disease, vestibular schwannoma, labyrinthitis, etc.). UVH was defined as a unilateral vestibular deficit, which could vary from (relatively) mild hypofunction to areflexia. UVH needed to be demonstrated by caloric test and/or, rotatory chair test, and/or (video) head impulse test. Therefore, studies were excluded in case none of these vestibular tests were performed. Studies investigating animals and/or patients with central vestibular pathologies and bilateral vestibulopathy were also excluded. Regarding outcomes, only studies with chronic UVH symptoms were selected. Chronic symptoms were defined as symptoms that lasted ≥3 months, according to the clinical practice guideline from the academy of neurologic physical therapy of the American physical therapy association (33). Both self-reported symptoms and self-report questionnaires were included for analysis.

### Quality assessment

The Quality in Prognosis Studies (QUIPS) tool was used to assess risk of bias. The level of evidence was graded using the EBRO-platform (Evidence-Based Guideline Development) (34). Level A1, A2, B, C, or D could be given, based on different items such as the number of participants and statistical power (Supplementary Table S4). Both reviewers independently assessed the risk of bias and level of evidence assessment and discussed the results during a consensus meeting. A total risk of bias score for each study was determined based on the guideline of the checklists: “low risk of bias,” “uncertain risk of bias,” or “high risk of bias.” Four types of biases were taken into consideration:

- *Selection bias*: “Low risk of bias” in case diagnostic criteria were clearly reported, “uncertain risk of bias” in case diagnostic criteria were not clearly reported.
- *Attrition bias*: “Low risk of bias” in case the drop-out rate was below 20%, “uncertain risk of bias” in case the drop-out rate was not reported, and “high risk of bias” in case the drop-out rate was above 20%.
- *Detection bias*: “Low risk of bias” in case valid questionnaires were reported, “uncertain risk of bias” in case questionnaires and self-reported symptoms were reported, “high risk of bias” in case only self-reported symptoms were reported.
- *Publication bias*: “Low risk of bias” in case the statistical analysis was clearly reported, “uncertain risk of bias” in case the statistical analysis was not clearly reported, “high risk of bias” in case the statistical analysis was not appropriate for the design of the study.

Interrater reliability was evaluated based on Cohen’s kappa. Cohen indicated that the kappa statistic can be categorized as no agreement (values <0), none to slight (0.01–0.20), fair (0.21–0.40), moderate (0.41–0.60), substantial (0.61–0.80), and almost perfect agreement (0.81–1.00). Four different questions indicating four different biases were assessed by Cohen’s kappa: Selection, attrition, detection, and publication bias.

### Data extraction

The relevant data were extracted from the included articles by both reviewers (see Supplementary Tables S5, S6, for further details). First, patient characteristics were extracted, which comprised: total number of patients, age, distribution of gender, etiology, diagnosis, and duration of symptoms. Secondly, all symptoms possibly related to UVH were extracted (self-reported and self-report questionnaires). However, symptoms clearly not related to the vestibular deficit, but related to etiology (e.g., hearing loss after intratympanic gentamicin) were not selected. Thirdly, diagnostic tests and their criteria used for diagnosing UVH (caloric test, rotatory chair test, (video) head impulse test), were extracted. Finally, for self-reported questionnaires, means and standard deviations were extracted (for pre-and post-intervention assessment in studies investigating interventions) where possible.

### Data synthesis

The main outcome measure of this systematic review was a detailed overview of chronic symptoms related to UVH, as reported in the literature. This included self-reported symptoms and self-report questionnaires. The self-reported symptoms were reviewed by three authors (MK, LVL, RvdB). In consensus, these self-reported descriptions were categorized using the universal symptom classification established by Lucieer et al. in patients with a bilateral vestibulopathy (35). The self-report questionnaires DHI, VSS, and VAS were included in the analysis, since these questionnaires were most frequently used in the selected studies. For description of patient characteristics, it was found that etiologies were defined differently between studies. Etiologies were therefore categorized into different entities, e.g., infectious/inflammatory, neoplasm, iatrogenic, vascular, and trauma (see Supplementary Table S5, 5th and 6th column). In case etiologies were not mentioned in a study, it was categorized as “missing.”

### Statistical analysis

Regarding self-reported symptoms, descriptive statistics (number and percentage) were used to show the frequency of each self-reported symptom. The percentage of each self-reported symptom was calculated by the total number of patients who reported the specific symptom (summed over all studies that assessed this symptom), divided by the number of patients that were included in the same studies, multiplied by 100%. It was decided to not divide by the total number of patients included in all studies, since patients did not have the opportunity to report on specific symptoms in all studies. Regarding the self-reported questionnaires, the means for each questionnaire (i.e., DHI, VSS, and VAS) were synthesized via meta-analysis using random-effects models fitted via restricted maximum-likelihood estimation. Based on the estimated pooled means and amount of heterogeneity as estimated from the random-effects models, we also estimated the percentage of true means for the DHI and VAS that are expected to fall above 30 and 3, respectively. For studies examining interventions, mean changes between the pre-and post-intervention assessment regarding the DHI and VAS scores were computed and were also synthesized using random-effects models. The correlation coefficient between the pre-and post-intervention assessments (which is needed to compute the sampling variances of the mean changes, but which is typically not reported) was approximated as 0.5, which could be considered a moderate correlation coefficient. For each model, we report the estimated pooled mean and mean change with a corresponding 95% confidence interval (CI). Besides, heterogeneity was analyzed using Cochran’s Q statistic, its degrees of freedom (df) and its corresponding value of *p*. Higgins’ I^2^ (%) was measured to assess the amount of heterogeneity that could be explained by true, i.e., between study variation. The interpretation of the amount of observed heterogeneity in Higgins’ bench-marking values was performed as around 25% (low heterogeneity), around 50% (moderate heterogeneity) and around or above 75% (high heterogeneity). Forest plots were used to visualize the results. The statistical analyses were carried out using R (version 4.2.2) (36) and the metafor package (version 3.8.1) (37).

## Results

### Study selection

**Figure 1:**
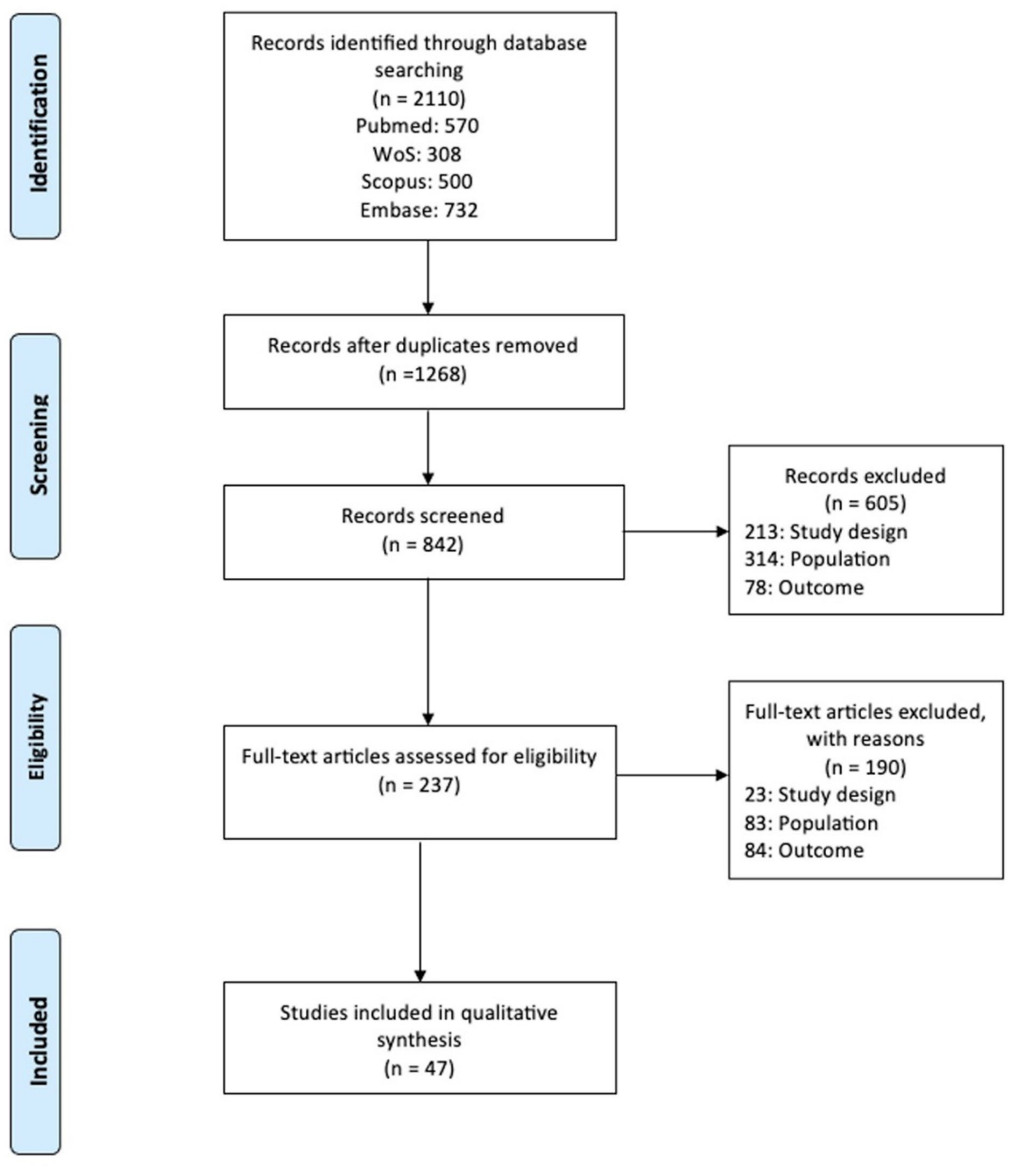
Flow chart of study selection.

A total of 2,110 articles were retrieved. After removing duplications (1,268 studies) via Endnote, a total of 842 citations were screened on title and abstract. After the second screening phase, 47 articles (2, 14, 16, 17, 24, 38–79) met the in-and exclusion criteria (Figure 1).

### Patient characteristics

A total of 1,478 adults, 799 females (54%) and 679 males (46%), were described, with study sample sizes ranging from 1 to 174. Study participants were between 18 and 84 years of age. The duration of symptoms ranged from at least 3 months to 42 years. A more detailed overview of patient characteristics can be found in Supplementary Table S5.

### Etiologies

Eight different etiology categories were identified in this systematic review, which could lead to UVH. The most frequent etiologies were Menière’s disease (38%) and infectious/inflammatory (16%). In 33% of the patients, the etiology was not described and therefore labeled as “missing” (Table 1).

**Table 1:** Etiologies of unilateral vestibular hypofunction reported in the studies included in this systematic review.

Etiologies (*n* = 1,461 patients)	Percentage
Menière’s disease	38%
Infectious/Inflammatory	16%
Neoplasm	3%
Idiopathic	3%
Iatrogenic	2%
Vestibular migraine	2%
Vascular	2%
Trauma	1%
Missing	33%

### Self-reported symptoms

A spectrum of self-reported symptoms related to UVH was reported in 37 studies. Chronic dizziness and imbalance were most reported. The self-reported chronic UVH symptoms were: chronic dizziness (98%), imbalance (81%), symptoms worsened by head movements (75%), visually induced dizziness (61%), symptoms worsened in darkness (51%), and oscillopsia (22%). Together with these symptoms, UVH could be accompanied by recurrent vertigo (77%), tiredness (68%), cognitive symptoms (58%), and autonomic symptoms (46%). Four studies reported additional symptoms beyond vestibular and hearing deficits such as headaches, ear/neck/back pain, limited social activities, and reduced quality of life (Table 1; Supplementary Table S6).

### Symptoms in self-report questionnaires

**Figure 2:**
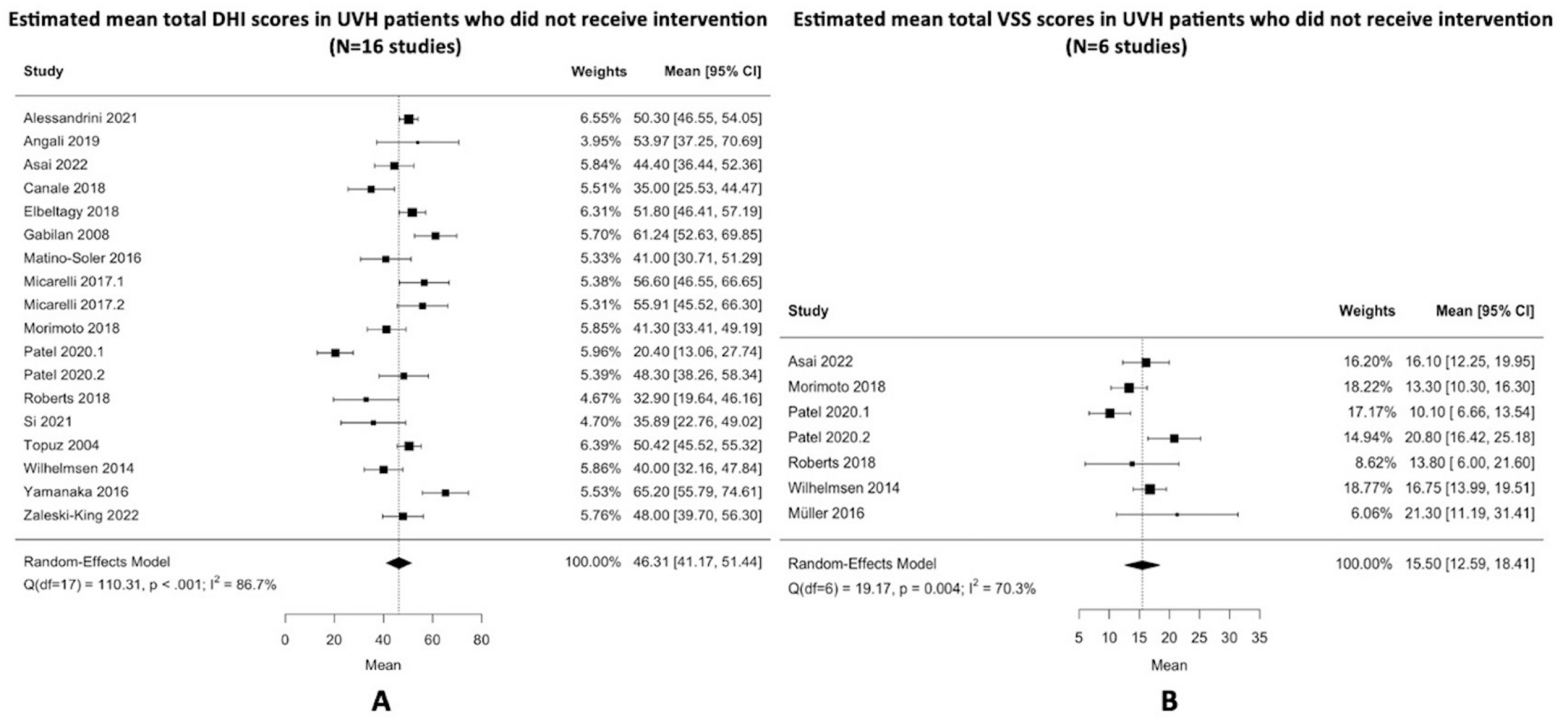
**(A,B)** Estimated mean total DHI and VSS scores in unilateral vestibulopathy patients who did not receive intervention. This includes DHI and VSS scores from studies without an intervention, as well as pre-intervention DHI and VSS scores from interventional studies. The dashed lines on the forest plots represent the overall pooled estimates. The black squares indicate the mean scores of each study and horizontal lines represent their 95% confidence intervals. The size of the black squares represents the weight contributed by each study in the meta-analysis. The black diamonds refer to the pooled odds ratio and their 95% confidence intervals. DHI, dizziness handicap inventory; VSS, vertigo symptom scale; Q, Cochran’s Q statistic; df, degrees of freedom; I^2^, Higgins’ calculation; CI, confidence interval.

Self-report questionnaires were administered in studies without and with interventions (different types of vestibular rehabilitation). Sixteen studies^1^ (2, 17, 24, 38–40, 43, 48, 62–65, 69, 74, 78, 79) investigated the total scores of the DHI and six studies (see footnote 1) (16, 24, 40, 64, 65, 69) investigated the total scores of the VSS in patients without (or before) intervention. The estimated mean total scores of the DHI and VSS were 46.31 (95% CI: 41.17–51.44) and 15.50 (95% CI: 12.59–18.41), respectively. These mean scores indicated a moderate handicap. The distributions of the means suggest that subgroups exist with little to no handicap, as well as with a severe handicap (see Figure 2).^2^ Additionally, while high and statistically significant heterogeneity was obtained between the studies that evaluated DHI (Q = 110.31; *p* < 0.001; I^2^ = 86.7%), moderate and statistically significant heterogeneity was found between the studies that evaluated VSS (Q = 19.17; *p* = 0.004; I^2^ = 70.3%).

**Figure 3:**
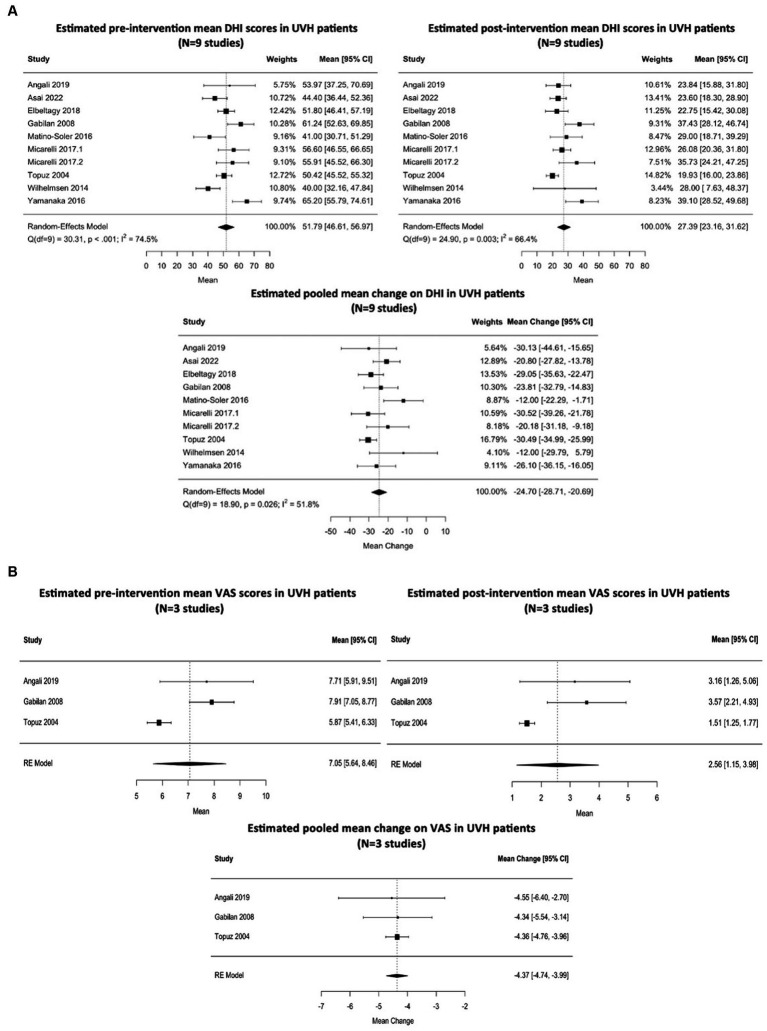
**(A)** Estimated mean DHI scores pre-intervention and post-intervention in patients with unilateral vestibulopathy (N = 9 studies). Interventions included vestibular rehabilitation therapy aiming to improve gaze stabilization and postural control, as well as providing coordination of sensorimotor strategies with active body control. The dashed line on the forest plots represents the overall pooled estimates. The black squares indicate the mean scores of each study and horizontal lines represent their 95% confidence intervals. The size of the black squares represents the weight contributed by each study in the meta-analysis. The black diamonds refer to the pooled odds ratio’s and their 95% confidence intervals. DHI, dizziness handicap inventory; Q, Cochran’s Q statistic; df, degrees of freedom; I^2^, Higgins’ calculation; CI, confidence interval. **(B)** Estimated mean VAS scores pre-intervention and post-intervention in patients with unilateral vestibulopathy (N = 3 studies). Interventions included vestibular rehabilitation therapy aiming to improve gaze stabilization and postural control as well as providing coordination of sensorimotor strategies with active body control. The dashed line on the forest plot represents the overall pooled estimate. The black squares indicate the mean scores of each study and horizontal lines represent their 95% confidence intervals. The size of the black squares represents the weight contributed by each study in the meta-analysis. The black diamonds refer to the pooled odds ratio and their 95% confidence intervals. VAS, visual analog scale; CI, confidence interval; RE, random-effects.

Nine studies (see footnote 1) (2, 24, 39, 40, 48, 62, 63, 74, 78) evaluated total DHI scores before and after an intervention. Interventions included vestibular rehabilitation therapy aiming to improve gaze stabilization, postural control, gait, and balance (N = 8 studies) as well as providing coordination of sensorimotor strategies with active body control (N = 1 study) (Supplementary Table S6). The estimated pooled pre-and post-intervention means on the DHI were 51.79 (95% CI: 46.61–56.97) and 27.39 (95% CI: 23.16–31.62), respectively. Heterogeneity between studies that evaluated DHI pre-intervention and post-intervention was moderate, respectively (Q = 30.31, I^2^ = 74.5%, *p* < 0.001; Q = 24.90, I^2^ = 66.4%, *p* = 0.003). The estimated pooled mean change based on the random-effects model was −24.70 (95% CI: −20.69 to −28.71). The percentage of true means that are expected to fall above 30 was estimated to be 32% in the post-intervention studies. A forest plot showing the observed outcomes and the estimate of the pooled mean DHI based on the random-effects model is shown in Figure 3A. Total VAS scores were compared in three studies (2, 38, 74) and the estimated pooled mean total VAS score decreased from 7.05 (95% CI: 5.64–8.46) (pre-intervention) to 2.56 (95% CI: 1.15–3.97) (post-intervention). The percentage of true means expected to fall above 3 was estimated to be 37% in the post-intervention studies. Only three studies evaluated VAS during pre-intervention and post-intervention, therefore heterogeneity assessments were not applied. A forest plot showing the observed outcomes and the estimate of the mean VAS based on the random-effects model is shown in Figure 3B.

### Risk of bias and level of evidence

Twenty-four out of the 47 articles were graded with a “low risk of bias,” while the remaining 23 were graded with an “unclear risk of bias.” The two main reasons for assigning an unclear risk of bias were uncertainty about vestibular testing diagnostic criteria and/or a dropout rate that was higher than 20%. No study seemed to have a high risk of bias. Regarding level of evidence, all interventional cross-sectional studies were graded with level of evidence B (*n* = 39) due to the type of study design. Only seven studies were considered as a level of evidence C, mostly due to the small sample size in these articles (See Supplementary Table S7). One study was classified as A2 due to the large sample size.

The Cohen’s kappa analysis revealed a substantial level of interrater reliability on all four risk of bias sections: selection bias (0.759), attrition bias (0.773), detection bias (0.709), and publication bias (0.754).

## Discussion

The aim of this systematic review was to investigate the full spectrum of chronic symptoms and the effects of interventions on these symptoms in patients with UVH. It was found that UVH can lead to a spectrum of symptoms, of which chronic dizziness (98%) and imbalance (81%) are most prevalent. Furthermore, vestibular rehabilitation can significantly decrease self-reported handicap in patients with chronic symptoms, but a subgroup of patients (≥32%) persists with at least a moderate handicap, despite vestibular rehabilitation.

Chronic dizziness and imbalance were most frequently reported and might be considered the main symptoms of UVH. Additionally, the spectrum of other symptoms related to UVH varied from, e.g., visually induced dizziness and oscillopsia, to tiredness and cognitive impairment. Oscillopsia was reported in 22% of the patients, which is lower than in patients with bilateral vestibulopathy (50–70%) (35). This indicates that one vestibular organ might often be sufficient to enable gaze stabilization and maintain dynamic visual acuity, but it can still fail in a subgroup of UVH patients. This again illustrates that maintaining dynamic visual acuity is a multifactorial process, in which the visual, oculomotor, and vestibular system are all involved: some systems might (partially) compensate for the unilateral loss of vestibulo-ocular reflex (80). Additionally, many of the self-reported symptoms are not specific for UVH. For instance, visually induced dizziness can be related to Persistent Postural Perceptional Dizziness (PPPD) (81). Furthermore, the high percentage of patients with head movement induced worsening of symptoms should be noted. After all in clinical practice, this symptom of hypofunction might often lead to a misdiagnosis of Benign Paroxysmal Positional Vertigo or cervical vertigo (82).

The additional symptoms of cognitive impairment, autonomic symptoms, and tiredness were only reported in six out of 46 studies. These symptoms were most likely underreported. After all, it could be hypothesized that in many studies these additional symptoms were not part of the standard history taking process and questionnaires, and patients might find it challenging to precisely describe their symptoms and to understand the interrelation of these symptoms with their vestibular deficit (82). In addition, acute (e.g., acute unilateral vestibulopathy/vestibular neuritis), episodic (e.g., Menière’s disease), and chronic vestibular disorders (e.g., vestibular schwannoma) might all lead to UVH symptoms. This complicates further history taking, since vertigo attacks can co-exist with UVH symptoms. Therefore, it could be advised to perform structured history taking in patients with UVH (22). Acronyms like SO STONED (83) and DISCOHAT (14) may be used to improve history taking and capture the symptoms related to UVH. For research purposes, semi-structured interviews should be conducted to define the whole spectrum of UVH symptoms more precisely. This should include a structural evaluation of DISCOHAT symptoms. In addition, open ended-questions and respondent-driven topics could be used to evaluate other symptoms related to UVH. This would facilitate the development of a validated PROM for UVH patients.

The high prevalence of chronic dizziness and imbalance, and the self-reported handicaps in UVH patients, illustrate that vestibular compensation and vestibular rehabilitation in the (sub)acute phase are not always sufficient to decrease symptoms, as previously described (9–12). After all, vestibular compensation is often sufficient for symptoms in static conditions, but not for symptoms in dynamic conditions (7). Unfortunately, current laboratory tests are not (yet) adequate to precisely determine the state of vestibular compensation (84). Therefore, it remains difficult to predict which UVH patients will keep on having (disabling) symptoms related to vestibular hypofunction. Nevertheless, vestibular rehabilitation still provides a substantial benefit in many patients, even though it is not globally unified, and a placebo-effect cannot always be ruled out. Depending on the type of program (office versus home-based), duration of intervention, protocol, applied equipment, and the provider of the training (ENT, physiotherapist, audiologist, etc.), as well as patient commitment, the outcomes might differ. However, vestibular rehabilitation should be offered to adults with UVH who present with symptoms, activity limitations, and participation restrictions as a result from UVH (21, 85, 86). According to the results found in this systematic review, a subgroup of patients does not sufficiently benefit from vestibular rehabilitation and maintains a moderate level of disability. In case the disability of these patients results from UVH and not from the primary and/or secondary pathology (e.g., Menière’s disease or Persistent Postural Perceptional Dizziness), not many additional interventions are currently clinically available. Therefore, it could be considered to explore new intervention strategies like noisy galvanic vestibular stimulation (87), vibrotactile feedback (88) or a vestibular implant (89). Especially for vestibular implantation, patient selection should be carefully investigated: not all UVH patients with remaining symptoms after vestibular rehabilitation would be eligible for implantation (90).

Finally, it should be noted that emotional and environmental factors can influence the experienced symptoms of UVH (91, 92). This also (partially) explains why objective vestibular findings (e.g., laboratory test results) do not perfectly match with subjective vestibular complaints (e.g., symptoms captured with history taking and/or questionnaires) (93, 94). Therefore, it would be advised to screen patients with chronic symptoms of UVH for underlying psychological/psychiatric/functional disorders which might negatively influence their vestibular symptoms (22). Eventually, treatment of these co-morbidities might improve their experienced burden of disease.

### Limitations

Five limitations were identified when performing this systematic review. First, different diagnostic criteria were used to define UVH (e.g., different cut-off values for vestibular tests), which led to a relatively heterogeneous study population, varying from mild to severe UVH. Second, UVH symptoms were collected differently (e.g., history taking, self-report questionnaires) which could induce a selection bias, especially risking underreporting of symptoms (see above). Third, since patients find it difficult to reliably describe their symptoms (82), the same symptom type might be described differently by different patients (e.g., vertigo and dizziness may be used interchangeably in some languages). This could have resulted in the same symptom being classified into different symptom categories (Table 2). Fourth, vestibular compensation can take up to 1 year (7) and this systematic review included studies with UVH symptoms lasting (only) 3 months or longer. This might imply that the prevalence and reported burden of some UVH symptoms could be different after full vestibular compensation. However, since many studies included patients with symptoms lasting more than 1 year, this effect is not expected to be substantial. Finally, in this systematic review, patients with UVH were included who could also have recurrent vertigo attacks and/or psychological comorbidities. This may have resulted in moderate to high heterogeneity in studies that evaluated self-reported questionnaires. It should be pointed out that not only UVH influences questionnaire scores or duration of symptoms, but also the primary pathology (e.g., Menière’s disease) and/or secondary pathology (e.g., Persistent Postural Perceptional Dizziness). It is therefore imperative to treat the presence of current attacks and/or psychological comorbidity together with UVH symptoms (22).

**Table 2:** Symptoms of patients with unilateral vestibular hypofunction, as reported in the studies included in this review.

Symptoms	Number of patients included for analysis	Reported (%)
Chronic dizziness	528	98%
Imbalance	606	81%
Recurrent vertigo	572	77%
Head movements worsen symptoms	144	75%
Tiredness	149	68%
Visually induced dizziness	144	61%
Cognitive complaints	145	58%
Darkness worsens symptoms	161	51%
Autonomic complaints	165	46%
Oscillopsia	151	22%

## Conclusion

A spectrum of symptoms is associated with UVH, of which chronic dizziness and imbalance are most frequently reported. Other symptoms include, among others, visually induced dizziness, oscillopsia, autonomic complaints, cognitive complaints, and tiredness. However, semi-structured interviews should be conducted to define the whole spectrum of UVH symptoms more precisely, in order to establish a validated PROM for UVH patients. Furthermore, vestibular rehabilitation can significantly decrease self-reported handicap, but a subgroup of patients (≥32%) persists with at least a moderate handicap, despite vestibular rehabilitation. For this subgroup, it could be considered to explore new intervention strategies like noisy galvanic vestibular stimulation (87), vibrotactile feedback (88) or the vestibular implant (89).

## Data availability statement

The original contributions presented in the study are included in the article/ Supplementary material, further inquiries can be directed to the corresponding author.

## Author contributions

MK, LVL and RvdB created the concept and design. MK and LVL ensured data acquisition. MK and WV provided data analysis and/and interpretation. RvdB supervised the writing and edited the manuscript. AH, LV, VVR, WV, AMe, LS, AMo, AP, and NG reviewed the manuscript. All authors contributed to the article and approved the submitted version.

## Conflict of interest

The authors declare that the research was conducted in the absence of any commercial or financial relationships that could be construed as a potential conflict of interest.

## Publisher’s note

All claims expressed in this article are solely those of the authors and do not necessarily represent those of their affiliated organizations, or those of the publisher, the editors and the reviewers. Any product that may be evaluated in this article, or claim that may be made by its manufacturer, is not guaranteed or endorsed by the publisher.

## Footnotes

^1^Micarelli 2017 and Patel 2020 included two groups in their studies, in which DHI scores were separately reported. Those separate groups were therefore reported as Micarelli 2017–1, Micarelli 2017–2, Patel 2020–1, and Patel 2020–2 in the forest plots.

^2^In some studies, comparatively low standard deviations (SDs) were reported (17, 24, 39,48, 63), which raises the concern that those SDs are actually standard errors (SEs). We therefore repeated the analyses back-calculating the SDs from the suspected SEs. While heterogeneity was reduced, the impact on the pooled estimates was negligible.
