# Supplementary material for: Chronic symptoms in patients with unilateral vestibular hypofunction: systematic review and meta-analysis
Source: Front Neurol. 2023 Jul 7;14:1177314. doi: 10.3389/fneur.2023.1177314 (PMC10360052; doi:10.3389/fneur.2023.1177314)
Supplement: Supplementary file 1 [file Table_1.pdf]

Table S1: P(IC)O-strategy

Population<br><br>OR		Outcome<br><br>OR
Unilateral vestibular hypofunction		Chronic symptoms
Unilateral vestibular loss		Chronic signs
Unilateral vestibular failure	A	Chronic complaints
Unilateral vestibular dysfunction	N	Terms referring to chronicity:<br>- Persistent<br>- Enduring<br>- Permanent<br>- Uncompensated<br>- Continuous<br>- Recurrent
Unilateral vestibulopathy	D	
Unilateral vestibular deafferentation		
Unilateral vestibular disease		
Unilateral vestibular disorder		
Unilateral vestibular syndrome		

Creating (PIC)O- strategy using search terms based on Medical Subject Headings keywords

Table S2. Search queries for each database

**WEB OF SCIENCE**<br>N: 308 hits on 4th of November 2022<br>(((Symptom* OR Sign* OR Complaint*)) AND (((Chronic OR Uncompensated OR Persistent OR Enduring OR Permanent OR Recurrent OR Continuous)) AND ((Unilateral) AND (((Vestibular) AND ((Hypofunction OR Failure OR Loss OR Deafferentation OR Disease OR Disorder OR Syndrome OR Impairment OR Dysfunction))) OR (Vestibulopathy)))))
**EMBASE**<br>N: 732 hits on 4th of November 2022<br>(((symptom* OR sign* OR complaint*)) AND (((chronic OR uncompensated OR persistent OR enduring OR permanent OR recurrent OR continuous)) AND ((unilateral) and (((vestibular) AND ((hypofunction OR failure OR loss OR deafferentation OR disease OR disorder OR syndrome OR ımpairment OR dysfunction))) OR (vestibulopathy))))) ti, ab, kw.
**PUBMED**<br>N:570 hits on 4th of November 2022<br>(((Symptom*[All Fields] OR Sign*[All Fields] OR Complaint*[All Fields])) AND (((Chronic[All Fields] OR Uncompensated[All Fields] OR Persistent[All Fields] OR Enduring[All Fields] OR Permanent[All Fields] OR Recurrent[All Fields] OR Continuous[All Fields])) AND ((Unilateral[All Fields]) AND (((Vestibular[All Fields]) AND ((Hypofunction[All Fields] OR Failure[All Fields] OR Loss[All Fields] OR Deafferentation[All Fields] OR Disease[All Fields] OR Disorder[All Fields] OR Syndrome[All Fields] OR Impairment[All Fields] OR Dysfunction[All Fields]))) OR (Vestibulopathy[All Fields])))))
**SCOPUS**<br>N: 500 hits on 4th of November 2022<br>TITLE-ABS-KEY (((symptom* OR sign* OR complaint*)) AND (((chronic OR uncompensated OR persistent OR enduring OR permanent OR recurrent OR continuous)) AND ((unilateral) and (((vestibular) AND ((hypofunction OR failure OR loss OR deafferentation OR disease OR disorder OR syndrome OR ımpairment OR dysfunction))) OR (vestibulopathy)))))

Table S3. Inclusion and exclusion criteria

	Inclusion criteria	Exclusion criteria
**Study design**	– Randomized controlled trials (RCTs)<br>– Cohort studies<br>– Case control studies<br>– Case reports<br><br>Language: English, French, German	– Systematic reviews<br>– Meta-analyses<br>– Editorials<br>– Conference proceedings<br>– Letter to editors<br>– Abstract only<br>- Other languages
**Population**	- Unilateral Vestibular Hypofunction (UVH)<br><br>- Vestibular testing to determine UVH<br>• Caloric test and/or<br>• Rotatory chair test and/or<br>• (Video) head impulse test<br><br>**Age: ≥ 18 years old**	- Healthy subjects<br>- Bilateral vestibulopathy<br>- Central vestibular disorders<br>- Animal Studies<br><br>**Age: < 18 years old**
**Outcome**	- Self-reported Symptoms<br><br>- Patient-reported Questionnaires<br>• Dizziness Handicap Inventory (DHI)<br>• Vertigo Symptom Scale (VSS)<br>• Visual Analogue Scale (VAS)<br><br>- **Duration of symptoms ≥3 months**	- Only assessing physical signs and quality of life, no symptoms<br><br>- **Duration of symptoms < 3 months**

Table S4. Level of evidence, Evidence Guideline Development platform (EBRO platform (28))

Level of evidence	Interventional studies	Diagnostic accuracy studies	Harm, side effects, etiology and prognosis
Level A1	Systematic review/meta-analysis of at least two independently conducted studies of A2 level		
Level A2	Randomized, double blind trial with good study quality and an adequate number of study participants	*Index test compared to reference test (reference standard); cut-offs were defined a priori; independent interpretation of test results; an adequate number of consecutive patients were enrolled; all patients received both tests*	*Prospective cohort study of sufficient magnitude and follow-up, adequately controlled for 'confounding' and no selective follow-up*
Level B	Clinical trial, but without all the features mentioned for level A2 (including case-control study, cohort study)	*Index test compared to reference test, but without all the features mentioned for level A2*	*Prospective cohort study, but without all the features mentioned for level A2 or retrospective cohort study or case-control study*
Level C	Non-comparative studies		
Level D	Expert opinion		

Table S5. Data extraction: Patient characteristics of the studies included in this systematic review

Authors	Total Number of Patients	Gender	Age (Years)	Diagnosis/ Etiology/ N			Duration of Symptoms
Alessandrini 2021	46	19 Female<br>27 Male	Mean±SD:<br>51.7±9.3	Acute unilateral vestibulopathy/ Vestibular neuritis	Infectious/Inflammatory	23	Mean±SD:<br>16.3±7 (months)
				Vestibular schwannoma	Neoplasm	13	
				Previous otological surgery	Iatrogenic	8	
				Ramsay hunt syndrome	Infectious/Inflammatory	2	
Angali 2019	31	15 Female<br>16 Male	Range: 24-59<br>Mean±SD:<br>39.48±10.96	Unilateral vestibular hypofunction	Missing	31	Mean±SD:<br>4.58±2.28 (years)
Asai 2022	21	15 Female<br>6 Male	Mean±SD:<br>60.9±13.7	Unilateral vestibular hypofunction	Missing	21	Mean±SD:<br>17.9±14.2 (months)
Bamiou 2000	44	23 Female<br>21 Male	Range: 20-65<br>Median (IQR):<br>48.5 (39.25-57.5)	Idiopathic	Idiopathic	13	Range: 6 months- 5 years
				Migraine	Vestibular migraine	9	
				Vascular inner ear disease	Vascular	6	
				Viral labyrinthitis	Infectious/Inflammatory	5	
				Inner ear infection	Infectious/Inflammatory	4	
				Head trauma	Trauma	4	
				Ramsay hunt syndrome	Infectious/Inflammatory	3	
Binetti 2017	1	1 Female	30	Acute unilateral vestibulopathy/ Vestibular neuritis	Infectious/Inflammatory	1	>9 months
Canale 2018	15	8 Female<br>7 Male	Range: 24-65<br>Mean: 50.8	Menière's Disease<br>(Vestibular Neurectomy)	Menière's Disease	15	Range: 3-12 years<br>Mean:6.7 years
Casani 2005	26	13 Female<br>13 Male	Range: 38-80<br>Mean: 58	Menière's Disease<br>(Chemical labyrinthectomy)	Menière's Disease	26	Range: 20-151 months<br>Mean: 49 months
Cohen 2017	20	6 Female<br>14 Male	Mean± SD:<br>62.5±7	Unilateral vestibular hypofunction	Missing	20	≥3 months
Corna 2003	14	7 Female<br>7 Male	Mean±SD:<br>58.9±12.9	Vascular inner ear disease	Vascular	7	≥3 months
				Vestibular schwannoma	Neoplasm	3	
				Idiopathic	Idiopathic	2	
				Menière's Disease	Menière's Disease	1	
				Head trauma	Trauma	1	
Crane 2017	4	3 Female<br>1 Male	Range: 31-74<br>Mean: 51	Vestibular schwannoma	Neoplasm	2	Range: 3-17 months
				Acute unilateral vestibulopathy/ Vestibular neuritis	Infectious/Inflammatory	1	
				Labyrinthitis	Infectious/Inflammatory	1	
Elbeltagy 2018	20	12 Female<br>8 Male	Range: 20-60<br>Mean±SD:<br>41.25±6.47	Unilateral vestibular hypofunction	Missing	20	Range:<br>3-12 months: 13<br>>12 months: 7
Foster 1994	6	3 Female<br>3 Male	Range: 36-67<br>Mean±SD:	Menière's Disease<br>(Vestibular neurectomy: 4	Menière's Disease	5	Range: 4 months -9 years<br>Mean: 5.3 years

			50.83±1.41	Labyrinthectomy: 1)			
				Vestibular schwannoma	Neoplasm	1	
Fujimoto 2012	16	8 Female<br>8 Male	Range: 36-77<br>Mean±SD:<br>57.2±12.6	Vestibular schwannoma	Neoplasm	8	Range:<br>3-6 months: 6<br>6-12 months: 3<br>≥12 months: 7
				Acute unilateral vestibulopathy/<br>Vestibular neuritis	Infectious/Inflammatory	5	
				Labyrinthitis	Infectious/Inflammatory	2	
				Previous otological surgery	Iatrogenic	1	
Fujimoto 2013	1	1 Male	43	Unilateral vestibular hypofunction	Missing	1	7 months
Gabilan 2008	21	18 Female<br>3 Male	Range: 20-63	Menière's Disease	Menière's Disease	7	Range:<br>3 months-1 year:3<br>1-10 years: 13<br>10-20 years: 4<br>>20 years: 1
				Vascular inner ear disease	Vascular	5	
				Acute unilateral vestibulopathy/<br>Vestibular neuritis	Infectious/Inflammatory	2	
				Labyrinthitis	Infectious/Inflammatory	2	
				Vestibular schwannoma surgery	Neoplasm	2	
				Head trauma	Trauma	2	
				Idiopathic	Idiopathic	1	
Gamba 2022	48	24 Female<br>24 Male	Range: 58-87<br>Mean: 71	Unilateral vestibular hypofunction	Missing	48	≥6 months
Ghulyan-Bedikian 2013	43	25 Female<br>18 Male	Range: 38-84	Unilateral vestibular hypofunction	Missing	26	>1 year
				Menière's Disease<br>(Vestibular Neurectomy)	Menière's Disease	14	
				Vestibular schwannoma	Neoplasm	3	
Gill-Body 1994	1	1 Female	62	Acute unilateral vestibulopathy/<br>Vestibular neuritis	Infectious/Inflammatory	1	6 months
Giray 2009	20	14 Female<br>6 Male	Range: 26-78	Unilateral vestibular hypofunction	Missing	20	≥3 months
Henriksson 2011	14	10 Female<br>4 Male	Mean±SD:<br>73.6±1.6	Acute unilateral vestibulopathy/<br>Vestibular neuritis	Infectious/Inflammatory	9	>1 year
				Labyrinthitis	Infectious/Inflammatory	3	
				Menière's Disease	Menière's Disease	2	
Kirazli 2020	10	6 Female<br>4 Male	Range: 34-65<br>Mean±SD:<br>50.30± 10.02	Menière's Disease	Menière's Disease	7	Range: 4-120 months<br>Mean±SD:<br>45.5±38.95 months
				Acute unilateral vestibulopathy/<br>Vestibular neuritis	Infectious/Inflammatory	3	
Kitahara 2018	60	34 Female<br>26 Male	Mean±SD:<br>46.2±15.1	Menière's Disease	Menière's Disease	60	Mean±SD:<br>48.8±30.6 months
Lazaro 2008	1	1 Male	48	Menière's Disease	Menière's Disease	1	3 years
Lopez 2007	40	20 Female<br>20 Male	Range: 22-74<br>Mean±SD: 48±13	Menière's Disease	Menière's Disease	40	Range: 1-22 years<br>Mean±SD:<br>6±6 years
Martin 2003	71	35 Female<br>36 Male	Mean: 53.6	Menière's Disease<br>(Chemical labyrinthectomy)	Menière's Disease	71	Mean: 12.6 months
Matino-Soler 2016	16	9 Female<br>7 Male	Range: 32-79<br>Mean±SD: 55±13	Acute unilateral vestibulopathy/<br>Vestibular neuritis	Infectious/Inflammatory	9	Range: 6-15 month<br>Mean±SD:<br>9.2±4.6 months
				Vestibular schwannoma	Neoplasm	4	
				Menière's Disease<br>(Chemical labyrinthectomy)	Menière's Disease	3	

Micarelli 2017	47	20 Female<br>27 Male	Head mounted display (HMD) +Vestibular Rehabilitation (VR) Group:<br>Mean±SD:<br>49.72±10.34	Acute unilateral vestibulopathy/ Vestibular neuritis	Infectious/Inflammatory	14	Mean±SD:<br>9.91±2.15 months
				Vestibular schwannoma	Neoplasm	4	
				Previous otological surgery	Iatrogenic	4	
				Ramsay hunt syndrome	Infectious/Inflammatory	1	
			Only VR group:<br>Mean±SD:<br>50.48±9.12	Acute unilateral vestibulopathy/Vestibular neuritis	Infectious/Inflammatory	13	Mean±SD:<br>9.37±1.55 months
				Vestibular schwannoma	Neoplasm	5	
				Previous otological surgery	Iatrogenic	4	
				Ramsay hunt syndrome	Infectious/Inflammatory	2	
Morimoto 2018	28	16 Female<br>12 Male	Mean±SD:<br>63.5±15.6	Unilateral vestibular hypofunction	Missing	28	Mean±SD:<br>18.1±14.6 months
Müller 2016	13	4 Female<br>9 Male	Range: 29-70<br>Mean±SD:<br>54.6±12.5	Acute unilateral vestibulopathy/ Vestibular neuritis	Infectious/Inflammatory	13	Range: 3-60 months<br>Mean±SD:<br>16.5±15.1
Paredis 2021	143	86 Female<br>58 Male	Range: 18-84<br>Mean:59	Menière's Disease	Menière's Disease	50	Range: 1-42 years<br>Mean±SD:<br>7±8 years
				Acute unilateral vestibulopathy/Vestibular neuritis	Infectious/Inflammatory	31	
				Idiopathic	Idiopathic	22	
				Vestibular migraine	Vestibular migraine	13	
				Previous otological surgery	Iatrogenic	8	
				Vascular inner ear disease	Vascular	6	
				Labyrinthitis	Infectious/Inflammatory	4	
				Head trauma	Trauma	4	
				Vestibular schwannoma	Neoplasm	3	
				Scleroderma	Auto-immune	1	
				Other	Other	1	
Patel 2020	35	10 Female<br>25 Male	Vestibular Neuritis<br>Mean±SD:<br>54.8±14.4<br>Menière's Disease<br>Mean±SD:<br>48.9±12.3	Acute unilateral vestibulopathy/ Vestibular neuritis	Infectious/Inflammatory	20	Range: 6-40 months
				Menière's Disease	Menière's Disease	15	>6 months
Perez 2003	71	35 Female<br>36 Male	Mean: 53.6	Menière's Disease<br>(Chemical labyrinthectomy)	Menière's Disease	71	Mean: 6.9 years
Quaglieri 2014	174	82 Female<br>92 Male	Mean±SD:<br>53.8±13.1	Menière's Disease<br>(Chemical labyrinthectomy)	Menière's Disease	174	Range: 6-90 months<br>Mean: 24 months
Rinaudo 2019	1	1 Female	58	Labyrinthitis	Infectious/Inflammatory	1	>3 years
Roberts 2018	17	9 Female<br>8 Male	Mean±SD:<br>58.8±17.3	Acute unilateral vestibulopathy/ Vestibular neuritis	Infectious/Inflammatory	17	>6 months
Sadeghi 2019	16	5 Female<br>11 Male	Range: 25-64<br>Mean±SD:<br>43.2±17.0	Unilateral vestibular hypofunction	Missing	16	Range: 1-8 years<br>Mean±SD: 3.5±2
Shotton 1989	6	5 Female<br>1 Male	Range: 33-66<br>Mean: 49.9	Menière's Disease<br>(Vestibular neurectomy)	Menière's Disease	6	Range: 2.7-5 years

Si 2021	18	12 Female<br>6 Male	Mean±SD:<br>47.94±11.01	Acute unilateral vestibulopathy/ Vestibular neuritis	Infectious/Inflammatory	18	Range: 5-72 months<br>Mean±SD: 18.61±2.82
Smółka 2020	58	43 Female<br>15 Male	Range: 40-64	Unilateral vestibular hypofunction	Missing	58	Range: >4 months
Tekin Dal 2021	75	40 Female<br>35 Male	Group 1:<br>Median (IQR):<br>38 (33-52)<br>Group 2:<br>Median (IQR):<br>42 (32-56)<br>Group 3:<br>Median (IQR):<br>43 (33-56)	Unilateral vestibular hypofunction	Missing	75	≥3 months
Topuz 2004	93	61 Female<br>32 Male	Range: 18–75<br>Mean±SD:<br>44.08±14.30	Unilateral vestibular hypofunction	Missing	93	Range: 6-46 months<br>Mean±SD: 15.76 ± 7.67
Ushio 2007	13	7 Female<br>6 Male	Range: 32-90	Acute unilateral vestibulopathy/ Vestibular neuritis	Infectious/Inflammatory	9	>6 months
			Mean: 73.3	Unilateral vestibular hypofunction	Missing	4	
Waterston 2004	3	3 Female	Range: 45-49<br>Mean±SD:<br>47±2.82	Unilateral vestibular hypofunction	Missing	3	Range: 6-12 months
Wilhelmsen 2014	3	2 Female<br>1 Male	Range: 56-73	Acute unilateral vestibulopathy/ Vestibular neuritis	Infectious/Inflammatory	3	Range: 9-29 months
Yagi 2021	10	3 Female<br>7 Male	Mean±SD:<br>65.7±8.1	Unilateral vestibular hypofunction	Missing	10	≥3 months
Yamanaka 2016	16	11 Female<br>5 Male	Range: 29-79<br>Mean: 59.8	Acute unilateral vestibulopathy/ Vestibular neuritis	Infectious/Inflammatory	NR	>5 years
				Ramsay hunt syndrome	Infectious/Inflammatory	NR	
				Labyrinthitis	Infectious/Inflammatory	NR	
				Vestibular schwannoma surgery	Neoplasm	NR	
Zaleski-King 2022	27	15 Female<br>12 Male	Range: 18-70<br>Mean±SD:<br>48±0.05	Acute unilateral vestibulopathy/ Vestibular neuritis	Infectious/Inflammatory	10	≥3 months
				Persistent postural perceptional dizziness		8	
				Vestibular migraine	Vestibular migraine	6	
				Bening paroxysmal positional dizziness (BPPV)		4	
				Other (intracranial hypertension, Chiari, idiopathic)	Idiopathic	4	
				Menière's Disease	Menière's Disease	3	
				Vestibular schwannoma	Neoplasm	1	
				**Subjects may have had more than one diagnosis**			
**Abbreviations**: IQR: Interquartile range, NR: Not reported, SD: Standart Deviation							

Table S6. Data extraction: Patient-reported questionnaire scores, self reported symptoms, and vestibular tests of the articles included in this systematic review

Authors	Method of collecting symptoms	Questionnaire Scores	Intervention	Self-reported vestibular symptoms (N/ N_{total})	Caloric Test	Rotatory Chair test	(v)HIT
Alessandrini 2021	Questionnaire-based	**DHI** (Total score: Mean±SD)<br>50.3±12.98 (No intervention)	N/A	N/A	Bithermal caloric test:<br>≥25% asymmetry rate in maximum slow phase velocity between two ears	N/A	N/A
Angali 2019	Questionnaire-based	**DHI** (Total score: Mean±SD)<br>53.97±8.53 (Pre-intervention)<br>23.84±4.06 (Post-intervention)<br>**VAS** (Mean±SD)<br>7.71±0.92 (Pre-intervention)<br>3.16±0.97 (Post-intervention)	VRT	N/A	Caloric test:<br>>25% asymmetry rate in maximum slow phase velocity between two ears	N/A	HIT:<br>Positive clinical HIT
Asai 2022	Questionnaire-based	**DHI** (Total score: Mean±SD)<br>44.4±18.6 (Pre-intervention)<br>23.6±12.4 (Post-intervention)<br>**VSS** (Mean±SD)<br>16.1±9.0 (Pre-intervention)<br>10.8±6.7 (Post-intervention)	Gait exercises	N/A	Bithermal caloric test:<br>≥25% asymmetry rate in maximum slow phase velocity between two ears	N/A	N/A
Bamiou 2000	Self reported<br><br>Questionnaire-based	**DHI** (Total score:Median±IQR)<br>36 (19-65.5) (No intervention)	N/A	Chronic dizziness (39/44)	Bithermal caloric test:<br>>10% asymmetry rate in maximum slow phase velocity between two ears	Rotatory chair test:<br>Directional preponderance in velocity stepping test	N/A
Binetti 2017	Self Reported<br><br>Questionnaire-based	**DHI** (Total score: Mean)<br>66 (Pre-intervention)<br>12 (Post-intervention)	VRT	Imbalance (1/1)<br>Oscillopsia (1/1)<br>Chronic dizziness (1/1)<br>Darkness (1/1)	Caloric test:<br>78% asymmetry rate in maximum slow phase velocity between two ears	N/A	vHIT:<br>Unilateral horizontal canal gain< 0.8<br>The presence of compensatory saccades including covert and overt
Canale 2018	Questionnaire-based	**DHI** (Total score: Mean±SD)<br>35±18.72 (No intervention)	N/A	N/A	Bithermal caloric test:<br>≥20 % asymmetry rate in maximum slow phase velocity between two ears	N/A	vHIT:<br>Unilateral horizontal canal gain< 0.8<br>Unilateral vertical canal gain< 0.7
Casani 2005	Self reported	N/A	N/A	Recurrent vertigo (26/26)	Bithermal caloric test:<br>>20% asymmetry rate in maximum slow phase velocity between two ears	N/A	N/A
Cohen 2017	Self reported	N/A	N/A	Chronic dizziness (20/20)	Caloric test:<br>>60% asymmetry rate in maximum slow phase velocity between two ears	N/A	N/A
Corna 2003	Self reported	N/A	N/A	Imbalance (14/14)	Monothermal caloric test:<br>>20% asymmetry rate in maximum slow phase velocity between two ears (I looked at the reference of this article)	N/A	N/A

Crane 2017	Self reported<br><br>Questionnaire-based	**DHI** (Total score: Mean)<br>42 (Pre-intervention)<br>11.5 (Post intervention)	VRT	Chronic dizziness (1/4)<br>Imbalance (1/4)	Caloric test:<br>Caloric areflexia on one side.<br>Ice water caloric:<br>Caloric areflexia on one side.	N/A	N/A
Elbeltagy 2018	Self-reported<br><br>Questionnaire-based	**DHI** (Total score: Mean±SD)<br>51.80±2.75 (Pre-intervention)<br>22.75±3.74 (Post intervention)	VRT	Chronic dizziness (20/20)	Bithermal caloric test:<br>>25% asymmetry rate in maximum slow phase velocity between two ears	N/A	N/A
Foster 1994	Self-reported	N/A	N/A	Oscillopsia (6/6)<br>Recurrent vertigo (2/6)	Ice caloric test:<br>Caloric areflexia on one side.	N/A	N/A
Fujimoto 2012	Self-reported	N/A	N/A	Recurrent vertigo (16/16)	Ice caloric test:<br>Caloric areflexia on one side.	N/A	N/A
Fujimoto 2013	Self-reported	N/A	N/A	Imbalance (1/1)	Ice caloric test:<br>>20% asymmetry rate in maximum slow phase velocity between two ears	N/A	N/A
Gabilan 2008	Questionnaire-based	**DHI** (Total score: Mean±SD)<br>61.24±20.12 (Pre-intervention)<br>37.43±21.77 (Post-intervention)<br>**VAS** (Total score:Mean±SD)<br>7.91±2.02 (Pre-intervention)<br>3.57±3.19 (Post-intervention)	VRT	N/A	Bithermal caloric test:<br>>25% asymmetry rate in maximum slow phase velocity between two ears	N/A	N/A
Gamba 2022	Self-reported	N/A	N/A	Chronic dizziness (48/48)<br>Imbalance (48/48)	N/A	N/A	vHIT:<br>Unilateral horizontal canal gain< 0.8
Ghulyan-Bedikian 2013	Self-reported<br><br>Questionnaire-based	**DHI**<br>N/A	N/A	Chronic dizziness (43/43)<br>Imbalance (43/43)	Bithermal cloric test:<br>≥34 asymmetry rate in maximum slow phase velocity between two ears	Rotatory chair test:<br>VOR gain≥ 0.4 at 0.25 Hz	N/A
Gill-Body 1994	Self-reported<br><br>Questionnaire-based	**DHI** (Total score:Mean)<br>14 (Pre-intervention)<br>NR (Post-intervention)<br>**VAS** (Total score:Mean)<br>4 (Pre-intervention)<br>3 (Post-intervention)	VRT	Imbalance (1/1)	Bithermal cloric test:<br>87% asymmetry rate in maximum slow phase velocity between two ears	Rotatory chair test:<br>Mildly decreased VOR gain at frequencies from 0.01 to 0.1 Hz	N/A
Giray 2009	Questionnaire-based	**DHI** (Total score:Median±IQR)<br>64.00 (30-92) (Pre-intervention)<br>22.00 (0-84) (Post-intervention)<br>**VAS** (Total score:Median±IQR)<br>4.45 (1.0-9.2)(Pre-intervention)<br>1.35 (0-7.1) (Post-intervention)	VRT	N/A	Bithermal caloric test:<br>>25% asymmetry rate in maximum slow phase velocity between two ears	N/A	N/A
Henriksson 2011	Self-reported	N/A	N/A	Imbalance (14/14)	Caloric test:<br>Reduced caloric response from the SCC on one side/ear.	N/A	HIT:<br>The lack of gaze stabilization indicating reduced vestibular function for the ear ipsilateral to the head thrust

Kirazli 2020	Self-reported	N/A	N/A	Imbalance (10/10)	Bithermal caloric test:<br>Caloric areflexia on one side.	N/A	N/A
Kitahara 2018	Self-reported	N/A	N/A	Chronic dizziness (60/60)	Monothermal caloric test:<br>Unilateral maximum slow phase eye velocity ≤10 degrees/second	N/A	N/A
Lazaro 2008	Self-reported	N/A	N/A	Recurrent vertigo (1/1)<br>Imbalance (1/1)<br>Chronic dizziness (1/1)<br>Autonomic symptoms (1/1)	Bithermal caloric test:<br>88% asymmetry rate in maximum slow phase velocity between two ears	Rotatory chair test:<br>Decreased VOR gain at frequencies from 0.01 to 0.16 Hz	N/A
Lopez 2007	Self-reported	N/A	N/A	Recurrent vertigo (40/40)	Bithermal caloric test:<br>≥22% asymmetry rate in maximum slow phase velocity between two ears	N/A	N/A
Martin 2003	Self-reported	N/A	N/A	Recurrent vertigo (17/71)	Bithermal caloric test:<br>No specific criteria described<br><br>Ice caloric test:<br>Caloric areflexia on one side.	Rotatory chair test:<br>No specific criteria described	N/A
Matino-Soler 2016	Self-reported<br><br>Questionnaire-based	**DHI** (Total score:Mean±SD)<br>41±21(Pre-intervention)<br>29±21(Post-intervention)	VRT	Imbalance (16/16)	N/A	N/A	vHIT:<br>Horizontal canal gain< 0.8<br>The presence of compensatory saccades including covert and overt
Micarelli 2017	Questionnaire-based	**DHI** (Total score:Mean±SD)<br>HMD+VR group<br>56.6±5.13 (Pre-intervention)<br>26.08±2.92 (Post-intervention)<br>Only VR group<br>55.91±5.3 (Pre-intervention)<br>35.73±5.88 (Post-intervention)	VRT	N/A	Bithermal caloric test:<br>>25% asymmetry rate in maximum slow phase velocity between two ears	N/A	N/A
Morimoto 2018	Self-reported<br><br>Questionnaire-based	**DHI** (Total score:Mean±SD)<br>41.3±21.3 (No intervention)<br>**VSS** (Total score:Mean±SD)<br>13.3±8.1 (No intervention)	N/A	Chronic dizziness (28/28)	Bithermal caloric test:<br>>40% asymmetry rate in maximum slow phase velocity between two ears	N/A	N/A
Müller 2016	Questionnaire-based	**VSS** (Total score:Mean±SD)<br>21.3± 18.6 (No intervention)	N/A	N/A	N/A	N/A	vHIT:<br>Unilateral horizontal canal gain< 0.8<br>Unilateral vertical canal gain< 0.7<br>The presence of compensatory saccades including covert and overt
Paredis 2021	Self-reported	N/A	N/A	Darkness (79/144)<br>Imbalance (99/144)<br>Supermarket effect (88/144)<br>Cognitive deficits (83/144)<br>Oscillopsia (26/144)<br>Head movements (108/144)	Bithermal caloric test:<br>>25% asymmetry rate in maximum slow phase velocity between two ears	N/A	N/A

				Autonomic symptoms (65/144)<br>Tiredness (99/144)			
Patel 2020	Questionnaire-based	**DHI** (Total score:Mean±SD)<br>Vestibular Neuritis: 20.4±14.5 (No intervention)<br>Menière's Disease: 48.3±22.9 (No intervention)<br>**VSS** (Total score:Mean±SD)<br>Vestibular Neuritis: 10.1±6.8 (No intervention)<br>Menière's Disease: 20.8±10 (No intervention)	N/A	N/A	Bithermal caloric test:<br>>25% asymmetry rate in maximum slow phase velocity between two ears	N/A	N/A
Perez 2003	Self-reported<br><br>Questionnaire-based	**DHI** (Total score:Mean)<br>56.25 (Pre-intervention)<br>29.42 (Post-intervention)	N/A	Imbalance (17/71)<br>Recurrent vertigo (17/71)	Bithermal caloric test:<br>>20% asymmetry rate in maximum slow phase velocity between two ears	Rotatory chair test:<br>Sinusoidal harmonic acceleration and velocity stepping tests were performed.	N/A
Quaglieri 2014	Self-reported	N/A	N/A	Recurrent vertigo (174/174)	Caloric test:<br>≥30% asymmetry rate in maximum slow phase velocity between two ears<br>Ice water caloric test:<br>Caloric areflexia on one side.	N/A	N/A
Rinaudo 2019	Self-reported<br><br>Questionnaire-based	**DHI** (Total score:Mean)<br>52 (Pre-intervention)<br>40 (Post-intervention)	VRT	Imbalance (1/1)<br>Tiredness (1/1)<br>Cognitive deficit (1/1)<br>Chronic dizziness (1/1)	Bithermal caloric test:<br>59% asymmetry rate in maximum slow phase velocity between two ears	N/A	N/A
Roberts 2018	Questionnaire- based	**DHI** (Total score:Mean±SD)<br>32.9±27.9 (No intervention)<br>**VSS** (Total score:Mean±SD)<br>13.8±16.4 (No intervention)	N/A	N/A	Bithermal caloric test:<br>≥20% asymmetry rate in maximum slow phase velocity between two ears	N/A	N/A
Sadeghi 2019	Self-reported	N/A	N/A	Imbalance (8/16)<br>Darkness (2/16)<br>Autonomic symptoms (8/16)	N/A	Rotatory chair test:<br>Directional preponderance > 10% at 0.2 Hz	N/A
Shotton 1989	Self-reported	N/A	N/A	Imbalance (1/6)	Bithermal Caloric test:<br>No specific criteria described	N/A	N/A
Si 2021	Self-reported<br><br>Questionnaire-based	**DHI** (Total score:Mean±SD)<br>35.89 ± 6.70 (No intervention)	N/A	Chronic dizziness (18/18)<br>Imbalance (18/18)	Bithermal caloric test:<br>>25% asymmetry rate in maximum slow phase velocity between two ears	N/A	N/A
Smółka 2020	Self-reported	N/A	N/A	Recurrent vertigo (58/58)<br>Imbalance (58/58)	Bithermal caloric test:<br>No specific criteria described	N/A	N/A

Tekin Dal 2021	Self-reported<br><br>Questionnaire-based	**VAS** (Total score: Median±IQR)<br>Group I<br>6 (6-7) (Pre-intervention)<br>5 (5-6) (Post-intervention)<br>Group 2<br>7 (6-7) (Pre-intervention)<br>6 (5-6) (Post-intervention)	Activity-based exercises<br><br>Cawthorne-Cooksey exercises	Chronic dizziness (75/75)	Bithermal caloric test:<br>>24% asymmetry rate in maximum slow phase velocity between two ears	N/A	N/A
Topuz 2004	Self-reported<br><br>Questionnaire-based	**DHI** (Total score:Mean±SD)<br>50.42 ± 24.124 (Pre-intervention)<br>19.93 ± 19.33 (Post-intervention)<br>**VAS** (Total score:Mean±SD)<br>5.87 ± 2.27 (Pre-intervention)<br>1.51± 1.29 (Post-intervention)	VRT	Chronic dizziness (93/93)<br>Imbalance (93/93)<br>Recurrent vertigo (93/93)	Bithermal caloric test:<br>>25% asymmetry rate in maximum slow phase velocity between two ears	N/A	N/A
Ushio 2007	Self-reported	N/A	N/A	Recurrent vertigo (13/13)<br>Chronic dizziness (13/13)	Monothermal caloric test:<br>>20% asymmetry rate in maximum slow phase velocity between two ears	N/A	N/A
Waterston 2004	Self-reported	N/A	N/A	Headache (3/3)<br>Autonomic symptoms (1/3)<br>Imbalance (1/3)<br>Recurrent vertigo (1/3)<br>Chronic dizziness (3/3)	Bithermal caloric test:<br>>25% asymmetry rate in maximum slow phase velocity between two ears<br><br>Directional preponderance >30%	Rotatory chair test:<br>Decreased VOR gain at frequencies from 0.1 to 0.4 Hz	N/A
Wilhelmsen 2014	Self-reported<br><br>Questionnaire-based	**DHI** (Total score: Mean±SD)<br>40.65±5.65 (Pre-intervention)<br>27.25±12.72 (Post-intervention)<br>**VSS** (Total score:Mean±SD)<br>16.75±1.41 (Pre-intervention)<br>10.25±10.60 (Post-intervention)	VRT	Headache (2/3)<br>Tiredness (1/3)<br>Cognitive symptoms (1/3)<br>Neck and back pain (1/3)<br>Ear pain (1/3)<br>Chronic dizziness (3/3)<br>Concentration difficulties (1/3)	Bithermal caloric test:<br>>25% asymmetry rate in maximum slow phase velocity between two ears	N/A	N/A
Yagi 2021	Self-reported	N/A	N/A	Chronic dizziness (10/10)	Bithermal caloric test:<br>>20% asymmetry rate in maximum slow phase velocity between two ears	N/A	vHIT:<br>Unilateral borizontal canal gain< 0.8<br>The presence of compensatory saccades including covert and overt
Yamanaka 2016	Self-reported<br><br>Questionnaire-based	**DHI** (Total score:Mean±SD)<br>65.2 ± 4.8 (Pre-intervention)<br>39.1 ± 5.4 (Post-intervention)	Vibrotactile stimulation Substitution	Chronic dizziness (16/16)<br>Imbalance (16/16)	Ice water caloric test:<br>Maximum slow phase velocity <3 degree/second.	N/A	N/A
Zaleski-King 2022	Self-reported<br><br>Questionnaire-based	**DHI** (Total score:Mean±SD)<br>48±22 (No intervention)	N/A	Chronic dizziness (27/27)<br>Imbalance (27/27)	Bithermal caloric test:<br>≥20% asymmetry rate in maximum slow phase velocity between two ears	Sinusoidal harmonic acceleration:<br>Presence of low frequency phase lead at 0,01, 0,32 and 0.64 Hz	vHIT:<br>Unilateral borizontal canal gain< 0.8<br>The presence of overt saccades
**Abbreviations:** VRT: Vestibular Rehabilitation Therapy, vHIT: Video Head Impulse Test, DHI: Dizziness Handicap Inventory, VAS: Visual Analog Scale, VSS: Vertigo Symptom Scale, N/A: Not applicable, NR: Not reported							

Table S7. Risk of Bias evaluation with the 'Quality in Prognostic Studies tool' and the level of evidence

Authors	Selection Bias	Attrition Bias	Detection Bias	Publication Bias	Risk of Bias	Level of Evidence
Alessandrini 2021	+	+	+	+	Low RoB	B
Angali 2019	+	+	+	+	Low RoB	B
Asai 2022	+	+	+	+	Low RoB	B
Bamiou 2000	+	−	+	+	Low RoB	B
Binetti 2017	+	+	−	−	Unclear RoB	C
Canale 2018	+	+	+	+	Low RoB	B
Casani 2005	+	+	?	−	Unclear RoB	B
Cohen 2017	+	?	−	?	Unclear RoB	B
Coma 2003	+	+	?	+	Low RoB	B
Crane 2017	+	−	?	+	Unclear RoB	B
Elbeltagy 2018	+	+	?	+	Low RoB	B
Foster 1994	+	+	−	−	Unclear RoB	C
Fujimoto 2012	+	+	−	−	Unclear RoB	B
Fujimoto 2013	+	+	−	−	Unclear RoB	B
Gabilan 2008	+	+	+	+	Low RoB	B
Gamba 2022	+	+	−	−	Unclear RoB	B

Ghulyan-Bedikian 2013	+	+	−	−	Unclear RoB	B
Gill-Body 1994	+	+	?	−	Unclear RoB	B
Giray 2009	+	+	?	+	Low RoB	B
Guidetti 2008	+	+	+	+	Low RoB	B
Henriksson 2011	+	+	−	−	Unclear RoB	B
Kirazli 2020	+	+	+	+	Low RoB	B
Kitahara 2018	+	+	+	+	Low RoB	B
Lazaro 2008	+	−	−	−	Unclear RoB	C
Lee 2019	+	+	?	−	Unclear RoB	B
Lopez 2007	+	+	−	−	Unclear RoB	B
Martin 2003	?	+	−	−	Unclear RoB	B
Matino-Soler 2016	+	+	+	?	Low RoB	B
Micarelli 2017	+	+	+	+	Low RoB	B
Morimoto 2018	+	+	?	+	Low RoB	B
Müller 2016	+	+	+	+	Low RoB	B
Patel 2020	+	+	+	?	Low RoB	B
Paredis 2021	+	+	+	+	Low RoB	A2
Perez 2003	+	−	?	?	Unclear RoB	C

Quaglieri 2014	+	+	−	?	Low RoB	C
Rinaudo 2019	+	+	?	−	Low RoB	C
Roberts 2018	+	+	+	?	Low RoB	B
Sadeghi 2019	+	?	?	?	Unclear RoB	B
Shotton 1989	+	+	?	−	Unclear RoB	B
Smółka 2020	+	+	+	+	Low RoB	B
Tekin Dal 2021	+	+	?	?	Unclear RoB	B
Topuz 2004	+	−	+	+	Low RoB	B
Ushio 2007	+	+	−	−	Unclear RoB	B
Waterston 2004	+	+	?	−	Unclear RoB	B
Wilhelmsen 2014	+	+	?	−	Unclear RoB	C
Yamanaka 2016	+	+	−	−	Unclear RoB	B
Zaleski-King 2022	+	+	+	+	Low RoB	B

Selection Bias: The study sample represents the population of interest on key characteristics, sufficient to limit potential bias of the observed relationship between prognostic factor and outcome.
Attrition Bias: Loss to follow-up (from baseline sample to study population analyzed) is not associated with key characteristics (i.e., the study data adequately represent the sample) sufficient to limit potential bias to the observed relationship between prognostic factor and outcome.
Detection Bias: Outcome of interest is adequately measured in study participants to sufficiently limit potential bias.
Publication Bias: The statistical analysis is appropriate for the design of the study, limiting potential for presentation of invalid or spurious results.
Note: Although Quality in Prognostic Studies tool consists of six questions only four out of six questions were used to evaluate risk of bias as the other two questions did not reflect the aim of this systematic review.

+ **: Low risk of bias** ? **: Unclear risk of bias** − **: High risk of bias**
